# Replication of cowpox virus in macrophages is dependent on the host range factor p28/N1R

**DOI:** 10.1186/s12985-021-01640-x

**Published:** 2021-08-23

**Authors:** Daniel Bourquain, Livia Schrick, Bernd Karsten Tischer, Klaus Osterrieder, Lars Schaade, Andreas Nitsche

**Affiliations:** 1 Centre for Biological Threats and Special Pathogens 1, Robert Koch Institute, Seestraße 10, 13353 Berlin, Germany; 2 Institute of Virology, Department of Veterinary Medicine, Freie Universität Berlin, Robert-von-Ostertag-Str. 7–13, 14163 Berlin, Germany; 3 Department of Infectious Diseases and Public Health, Jockey Club College of Veterinary Medicine and Life Sciences, City University of Hong Kong, Kowloon, Hong Kong

**Keywords:** Orthopoxvirus, Cowpox virus, Host range, p28, RING finger

## Abstract

Zoonotic orthopoxvirus infections continue to represent a threat to human health. The disease caused by distinct orthopoxviruses differs in terms of symptoms and severity, which may be explained by the unique repertoire of virus factors that modulate the host’s immune response and cellular machinery. We report here on the construction of recombinant cowpox viruses (CPXV) which either lack the host range factor p28 completely or express truncated variants of p28. We show that p28 is essential for CPXV replication in macrophages of human or mouse origin and that the C-terminal RING finger domain of p28 is necessary to allow CPXV replication in macrophages.

**Supplementary Information:**

The online version contains supplementary material available at 10.1186/s12985-021-01640-x.

## Background

Virions of members within the family *Poxviridae* are large and complex, contain a double-stranded DNA genome of 130–375 kbp, and virus replication takes place in the cytosol [1]. Among poxviruses, especially the genus *Orthopoxvirus* (OPV) contains several important pathogens, and endemic cowpox viruses (CPXV) are the most common cause of zoonotic OPV infections in Europe and parts of northern and central Asia today [2, 3].

Understanding CPXV pathogenesis has shifted focus on the vast array of poxvirus-encoded accessory proteins which give each poxvirus its unique characteristics of immunomodulation and pathogenesis [4–7]. Among these, the so-called host range genes are supposed to be responsible for differences in tropism and host range between individual poxviruses [6–8].

The p28 protein of poxviruses belongs to the KilA-N/RING domain-containing p28/N1R protein family [6–8]. The p28 protein was first described as a virulence factor of ectromelia virus (ECTV) [9]. Disruption of the p28 gene abolished lethality of ECTV for susceptible mice, although it had no effect on virus replication in several other cell types, with the exception of mouse peritoneal macrophages [9, 10]. The ECTV p28 gene was found to be highly conserved, and orthologues of p28 are found in the genomes of most OPV, with the notable exception of several strains of vaccinia virus (VACV) [6, 9, 11]. Furthermore, orthologues of the p28 gene are also present in several other genera of the *Chordopoxvirinae* [6, 12].

The full-length p28 protein consists of 242 amino acids and combines an N-terminal KilA-N domain, which acts as DNA-binding domain [13], and a C-terminal RING domain [6]. The protein is translated in the early phase of viral replication, is present throughout the viral life cycle and localizes to cytoplasmic virus factories facilitated by its KilA-N domain [9, 10, 14]. The mechanism of how p28 contributes to viral host range is currently not understood. In vitro studies showed that the p28 proteins of VACV strain IHD-W, ECTV strain Moscow, variola virus strain Bangladesh-1975 and myxoma virus strain Lausanne possess E3 ubiquitin ligase activity which was attributed to the RING domain [15, 16]. Mutation of the RING domain stabilizes p28, indicating that p28 regulates itself via autoubiquitination and subsequent proteasomal degradation [14, 16]. Furthermore, p28 seems to be ubiquitinated by a yet unknown cellular ubiquitin ligase [14]. As the RING domain of p28 has been shown to be essential for the functionality of p28 as a virulence and host range factor of ECTV [9, 10], the mechanism of how p28 contributes to viral host range most likely depends on its function as a ubiquitin ligase. However, so far no cellular or viral substrates for p28-mediated ubiquitination despite p28 itself have been identified. Furthermore, the presence of genes encoding only KilA-N domains in other poxviruses makes it possible that this domain exerts an ubiquitin ligase-independent function that might contribute to host range and virulence [6].

Functionally, it has been speculated that p28 directs the ubiquitination and degradation of substrate host proteins which block viral replication through the induction of apoptosis [11, 16], as inhibition of apoptosis by p28 has been shown [17, 18]. Furthermore, as infection of murine macrophages with a p28 knockout ECTV resulted in a block of viral DNA replication and abortive infection following early viral gene expression [10], it was suggested that p28 might functionally compensate for an unknown cellular factor essential for viral DNA replication in macrophages.

In this study, we aimed to elucidate the significance of p28/N1R as a host range factor in CPXV. With regards to the importance of p28 for ECTV virulence, analysing the role of p28 in CPXV infection is of high interest concerning the understanding of CPXV pathogenesis. Therefore, we created recombinant CPXV which were either deficient in p28 expression or encoded mutant p28 proteins lacking a functional C-terminal RING domain. We show that CPXV lacking a functional p28 replicated less efficiently in macrophages of human or mouse origin, indicating that CPXV—like ECTV—is dependent on p28 to productively infect cells of the macrophage lineage. Given the importance of macrophages in containing systemic poxvirus dissemination [19], p28 may be important for CPXV virulence in vivo.

## Generation of a CPXV mutant lacking host range factor p28

To study the function of p28 as a host range factor of CPXV, we generated different p28 mutant viruses of CPXV strain Brighton Red (BR) (Additional file 1: Figure S1). This was done via Red recombination of a CPXV BR genome cloned into a bacterial artificial chromosome (BAC) [20] as described previously [21, 22]. A p28 knockout mutant was created by mutation of the start codon of p28 (CPXV-∆p28). Furthermore, two RING-finger knockout mutants of CPXV were created: one by insertion of stop codons ahead of the RING finger domain, truncating the p28 protein at amino acid position 184 (CPXV-p28(1-184)). This mutant resembles the truncated p28 homologue encoded by the prototypic VACV strain Western Reserve (VACWR011 and VACWR207). The second RING finger knockout mutant encodes a full-length p28 protein in which the critical cysteine residues at positions 197, 202 and 205 and the critical histidine at position 199 were substituted with alanine to destroy the structure of the RING finger domain (CPXV-p28∆RING). Revertant viruses harbouring a reconstituted wildtype p28 were generated from all recombinant BACs and were shown to replicate comparably to wild-type CPXV BR in all cell lines tested, which is important to exclude effects caused by bystander mutations (data not shown). All recombinant viruses were rescued on VeroE6 cells and propagated on HeLa cells. To proof that the recombinant viruses harboured the anticipated mutations, all recombinant viruses’ genomes were sequenced (Additional file 1).

## p28 is necessary for efficient CPXV replication in macrophage cell lines

**Fig. 1:**
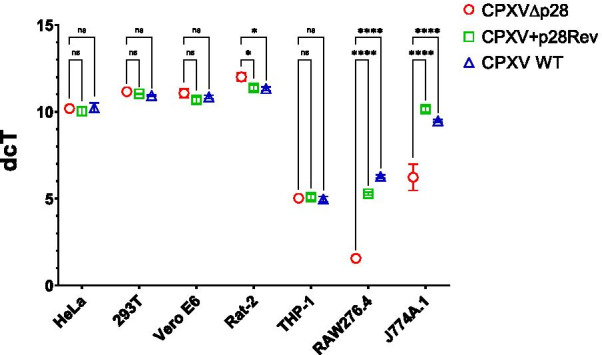
CPXV-∆p28 replication in different cell lines. Shown are ∆cT values (48 h p.i.) as a measure of viral DNA replication in cells infected (MoI = 0.1) with the p28 knockout virus CPXV-∆p28, the corresponding revertant virus and wild-type CPXV (n = 2). ∆cT values were obtained via normalization to expression of the cellular MYC gene. All cell lines were obtained from American Type Culture Collection (ATCC) and routinely screened for mycoplasma contamination. THP-1 cells were stimulated with PMA before infection. Statistics: two-way ANOVA and Tukey’s multiple comparison test

The replication competency of the p28 knockout mutant CPXV-∆p28 was analysed in different cell lines. Cells were infected with either CPXV-∆p28, the p28-expressing revertant (CPXV+p28-Rev) or wild-type (WT) CPXV BR at a multiplicity of infection (MoI) of 0.1. At 48 h post infection (p.i.), cells were lysed and viral genome copies were quantified via qPCR (Fig. 1) as described previously [23]. In HeLa, 293 T, Vero E6, Rat-2 and PMA-differentiated THP-1 cells, no differences between CPXV-∆p28 and the p28-expressing viruses could be observed. In contrast, CPXV-∆p28 replicated less efficiently than p28-expressing CPXV in the murine macrophage cell line J774A.1 and also slightly less efficiently in RAW267.4 macrophages.

## The host range effect of CPXV p28 in J774A.1 macrophages is dependent on the p28 RING domain

**Fig. 2:**
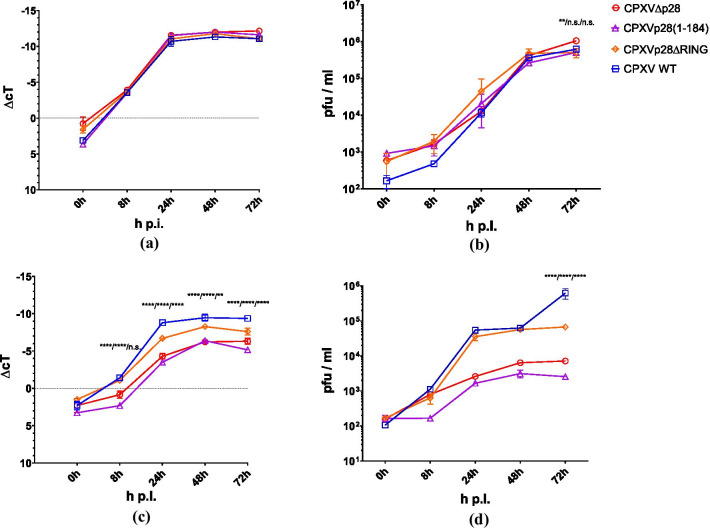
CPXV-∆p28, CPXV-p28(1-184) and CPXV-p28∆RING replication kinetics. The replication of CPXV in the Rat-2 fibroblast cells and J774A.1 mouse macrophages was analysed via qPCR and plaque assay (n = 2). Shown are ∆cT values as a measure of viral DNA replication in CPXV-infected (MoI = 0.1) **a** Rat-2 and **c** J774A.1 cells or infectious particles (pfu/ml) in the supernatant of **b** Rat-2 or **d** J774A.1 cells infected with the p28 mutant viruses CPXV-∆p28, CPXV-p28(1-184) and CPXV-p28∆RING. ∆cT values were obtained via normalization to expression of the cellular MYC gene. Statistics: two-way ANOVA and Tukey’s multiple comparison test. Asterisks showing significance of CPXVΔp28 vs. WT/CPXV p28(1-184) vs. WT/CPXVp28ΔRING vs. WT)

As CPXV-∆p28 showed reduced genome replication in J774A.1 cells, this cell line was chosen for more detailed analysis. Therefore, J774A.1 cells were infected with CPXV-∆p28, CPXV-p28(1-184), CPXV-p28∆RING as well as with the corresponding revertant viruses and wild-type CPXV. The replication competency of these viruses in J774A.1 cells was compared to virus replication in Rat-2 cells in which CPXV-∆p28 replicated efficiently. Cells were infected at an MoI of 0.1 and quantification of viral genome copies and infectious offspring viruses was done at 0 h, 8 h, 24 h, 48 h and 72 h p.i. via qPCR and plaque assay as described previously [24]. In Rat-2 fibroblast cells, all recombinant CPXV replicated as efficiently as the wild-type CPXV (Fig. 2). In contrast, CPXV-∆p28 and the RING mutants CPXV-p28(1-184) and CPXV-p28∆RING replicated less efficiently than wild-type CPXV in J774A.1 cells. This indicates that the host range function of CPXV p28 is dependent on the presence of a functional RING domain.

Similar results were obtained for p28 variations occurring naturally in different strains of VACV. We could show that only the full-length p28 encoding VACV strain IHD-W replicated efficiently in J774A.1 cells, whereas the replication of the VACV strain Western Reserve, which expresses a truncated p28 similar to p28(1-184) used in this study, was severely impaired (Additional file 1: Figure S2). Similarly, VACV strain Lister Elstree, which lacks most of p28, was unable to replicate in J774A.1 cells. However, the replication efficacy of VACV in macrophages may also be determined by further genetic differences between these strains of VACV, non-related to p28. Therefore, further research is needed to clarify the importance of p28 as a host range factor of VACV.

## p28 is essential for CPXV replication in primary rat macrophages and human PBMC-derived macrophages

**Fig. 3:**
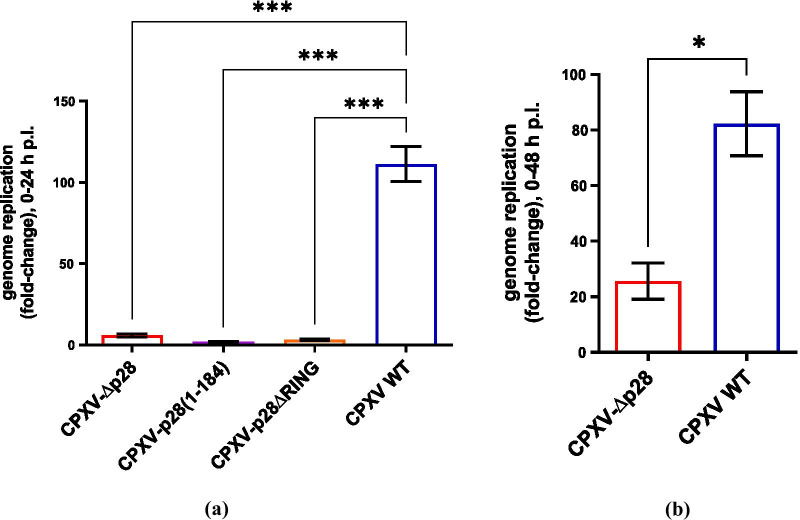
CPXV replication in primary macrophages. The replication of **a** CPXV-∆p28, CPXV-p28(1-184) in primary peritoneal rat macrophages and **b** of CPXV-∆p28 in human PBMC-derived GM-CSF-differentiated macrophages was analysed via quantification of viral genome replication in cell lysates (n = 2). Primary rat macrophages were isolated from the peritoneum of 3-month-old Wistar rats (Rattus norvegicus). PBMCs were isolated from fresh human buffy coat (acquired from DRK Blutspendedienst Ost) via Ficoll-Paque density gradient cell separation. Isolation of CD14+ monocytes was done via MACS separation using CD14+ MicroBeads (Miltenyi Biotec). For differentiation into macrophages, 100 ng/ml GM-CSF was added to the medium. Cells were infected with an MoI of 0.1 and viral genome copies in cell lysates were quantified via qPCR. Shown are fold-change values obtained via the ∆∆cT method after normalization of cT values to MYC gene expression. Statistics: **a** one-way ANOVA and Tukey’s multiple comparison test, **b** unpaired t-test

In addition to the macrophage cell line, we further analysed CPXV replication in primary peritoneal rat macrophages (Fig. 3a) and human PBMC-derived GM-CSF-stimulated macrophages (Fig. 3b). The cells were infected at an MoI of 0.1 and viral genome replication was assessed at 24 h and 48 h, respectively. Comparable to the murine J774A.1 macrophage cells, CPXV-∆p28, CPXV-p28(1-184) and CPXV-p28∆RING showed reduced genome replication in primary peritoneal rat macrophages. Similarly, a less efficient replication of CPXV-∆p28 was also observed in the human GM-CSF-differentiated PBMC-derived macrophages. However, CPXV replication of primary macrophages was overall much less efficient when compared to macrophage cell lines.

## Conclusion

Our results show that CPXV replication in cells of the macrophage lineage of human, mouse or rat origin is dependent on the presence of a functional p28 protein as has been described for ECTV [9, 10]. Furthermore, our results underline the importance of the ubiquitin ligase activity of p28 present within its C-terminal RING finger domain for p28 host range function. However, cellular or viral targets of ubiquitination via p28 remain unknown, as does the precise function of p28 in the infected cell. Further research will have to focus on the identification of p28 substrates to elucidate the role of p28 in virus virulence.

In the context of our previous results which show that CPXV infection—in contrast to VACV—induces a pronounced inflammatory response resulting in the attraction of leukocytes [25, 26], p28-mediated replication of CPXV in attracted macrophages may be of critical importance for CPXV dissemination and spread. The latter may be suggested by the important role that infected leukocytes are known to play in systemic OPV dissemination [27, 28]. Therefore, p28 may be important for CPXV virulence in vivo, as was shown to be for ECTV [9].

## Supplementary Information

**Additional file 1**. Mapping of the p28 loci from the sequenced recombinant viruses’ genomes to the CPXV BR WT p28 sequence derived from the CPXV BR reference sequence (NC_003663.2)

## Data Availability

Not applicable.

## Abbreviations

BAC: Bacterial artificial chromosome
BR: Brighton red
CPXV: Construction of recombinant cowpox viruses
ECTV: Ectromelia virus
GM-CSF: Granulocyte-macrophage colony-stimulating factor
MACS: Magnetic cell separation
MoI: Multiplicity of infection
OPV: *Orthopoxvirus*
p.i.: Post infection
PBMC: Peripheral blood mononuclear cell
qPCR: quantitative PCR
RING: Really interesting new gene
VACV: Vaccinia virus
