# Supplementary material for: Replication of cowpox virus in macrophages is dependent on the host range factor p28/N1R
Source: Virol J. 2021 Aug 23;18:173. doi: 10.1186/s12985-021-01640-x (PMC8381512; doi:10.1186/s12985-021-01640-x)
Supplement: Supplementary file 1 — Additional file 1. Mapping of the p28 loci from the sequenced recombinant viruses’ genomes to the CPXV BR WT p28 sequence derived from the CPXV BR reference sequence (NC_003663.2) [file 12985_2021_1640_MOESM1_ESM.docx]

**Figure S1.** Generation of recombinant viruses. Different p28 mutant viruses of CPXV strain Brighton Red (BR) were generated via Red recombination of a CPXV BR genome cloned into a bacterial artificial chromosome (BAC). A p28 knockout mutant was created by mutation of the start codon of p28 (CPXV-∆p28). A RING-finger knockout mutant was created by insertion of stop codons ahead of the RING finger domain, truncating the p28 protein at amino acid position 184 (CPXV-p28(1-184)). A second RING finger knockout mutant encodes a full-length p28 protein in which the critical cysteine residues at positions 197, 202 and 205 and the critical histidine at position 199 were substituted with alanine to destroy the structure of the RING finger domain (CPXV-p28∆RING). Revertant viruses encoding wildtype p28 were generated from all recombinant BACs via reversion of the inserted mutations. All virus genomes were sequenced for quality control.

**Figure S2.** Replication kinetics of different VACV strains in J774A.1 cells. The replication of VACV strains Lister Elstree (LE), Western reserve (WR) and IHD-W was analysed in J774A.1 mouse macrophages via qPCR (n = 4). Cells were infected with an MoI of 0.1. Shown are fold-change values obtained via the ∆∆cT method after normalisation of cT values to MYC gene expression. Statistics: One-way ANOVA and Tukey’s multiple comparison test.
